# SARS-Coronavirus-2 cases in healthcare workers may not regularly originate from patient care: lessons from a university hospital on the underestimated risk of healthcare worker to healthcare worker transmission

**DOI:** 10.1186/s13756-020-00848-w

**Published:** 2020-12-07

**Authors:** Sandra Schneider, Brar Piening, Pauline Assina Nouri-Pasovsky, Anne Caroline Krüger, Petra Gastmeier, Seven Johannes Sam Aghdassi

**Affiliations:** 1 Institute of Hygiene and Environmental Medicine, Charité-Universitätsmedizin Berlin, corporate member of Freie Universität Berlin, Humboldt-Universität zu Berlin, and Berlin Institute of Health, Berlin, Germany; 2 Department of Nephrology and Medical Intensive Care, Charité-Universitätsmedizin Berlin, corporate member of Freie Universität Berlin, Humboldt-Universität zu Berlin, and Berlin Institute of Health, Berlin, Germany

**Keywords:** SARS-Coronavirus-2, COVID-19, Outbreak, Healthcare-associated infection, Occupational health, Infection control

## Abstract

**Background:**

Coronavirus disease 2019 (COVID-19) caused by severe acute respiratory syndrome coronavirus 2 (SARS-CoV-2) represents an unprecedented healthcare challenge. Various SARS-CoV-2 outbreaks in healthcare facilities have been reported. Healthcare workers (HCWs) may play a critical role in the spread of the virus, particularly when asymptomatic. We examined four healthcare-associated outbreaks of SARS-CoV-2 infections that occurred at a university hospital in Berlin, Germany. We aimed to describe and analyze the spread of the virus in order to draw conclusions for effective containment of SARS-CoV-2 in healthcare facilities.

**Methods:**

Healthcare-associated outbreaks of SARS-CoV-2 infections were defined as two or more laboratory confirmed infections with SARS-CoV-2 where an epidemiological link within the healthcare setting appeared likely. We focused our analysis on one of three sites of the Charité-University Medicine hospital within a 2 month period (March and April 2020).

**Results:**

We observed four healthcare-associated outbreaks of SARS-CoV-2 infections, with a total of 24 infected persons (23 HCWs and one patient). The outbreaks were detected in the departments of nephrology and dialysis (n = 9), anesthesiology (n = 8), surgical pediatrics (n = 4), and neurology (n = 3). Each outbreak showed multiple unprotected contacts between infected HCWs. A combination of contact tracing, testing, physical distancing and mandatory continuous wearing of face masks by all HCWs was able to contain all four outbreaks.

**Conclusions:**

HCW to HCW transmission represented the likely source of the four outbreaks. Ensuring proper physical distancing measures and wearing of protective equipment, also when interacting with colleagues, must be a key aspect of fighting COVID-19 in healthcare facilities.

## Background

In December 2019, first reports emerged from Wuhan, China about a cluster of pneumonias with a suspected epidemiological link to a seafood wholesale market [1]. The underlying pathogen was identified and provisionally named 2019 novel coronavirus [2]. The virus was later renamed by the International Committee on Taxonomy of Viruses as severe acute respiratory syndrome coronavirus 2 (SARS-CoV-2) [3]. The disease caused by SARS-CoV-2 was named coronavirus disease 2019 (COVID-19) by the World Health Organization [4]. In late January 2020, the first infection with SARS-Cov-2 was detected in Germany, presumably linked to contact with a Chinese business traveler [5]. First cases of local transmission in Germany were noted in late February 2020 [6]. On 1 March 2020 the first case of COVID-19 in Berlin was confirmed [7]. Due to the accelerated spread of the disease, several measures were implemented in Germany, and Berlin specifically. Strict social distancing measures were set up and gatherings of more than two people were prohibited on 22 March 2020 [8]. Hospitals had to reduce the number of visitors to a minimum.

Droplets have been described as the main vector for human-to-human transmission of SARS-CoV-2 [9]. Asymptomatic infection and spread of the virus by pre-symptomatic or asymptomatic carriers have been reported in the early stages of the global outbreak [5]. Healthcare workers (HCWs) infected or colonized with pathogens pose a challenge in the practice of infection prevention and represent a potential source for healthcare-associated outbreaks [10, 11]. Multiple healthcare-associated clusters with SARS-CoV-2 have been reported [12, 13]. Commonly, infected patients are regarded as the biggest risk with regards to pathogen transmission for both other patients and HCWs. However, asymptomatically infected personnel can represent a complicating factor in the context of SARS-CoV-2 outbreaks in healthcare facilities [14, 15], and may be an underestimated risk for patient and HCW safety.

In this article, we report and examine four healthcare-associated outbreaks of SARS-CoV-2 infections that occurred at one of the sites of a university hospital in Berlin, Germany. We aim to describe and analyze the spread of the virus to draw conclusions for the effective containment of SARS-CoV-2 in healthcare facilities.

## Methods

Charité-University Medicine Berlin is a large tertiary care university hospital with three separate sites and more than 3000 beds. As a first response to the spread of the COVID-19 epidemic in China, the hospital required all return travelers from China to abstain from any activities at the hospital (i.e. patient care or any other kind of work at the hospital) and recommended isolation at home for 14 days. This regulation was put into effect on 14 February 2020, and subsequently extended to other risk areas (i.e. countries or other areas of the world with assumed widespread community-transmission of SARS-CoV-2 as defined by the German Robert Koch-Institute) as the spread of the disease continued. Reverse transcriptase polymerase chain reaction (RT-PCR) testing for SARS-CoV-2 was offered to all return travelers and explicitly recommended for those showing signs of infection. On 1 March 2020 the first COVID-19 patient was hospitalized at Charité’s Virchow campus. Personal protective equipment (PPE) recommended by Charité’s infection prevention and control (IPC) team for the care of COVID-19 patients consisted of a medical face mask, gloves and gowns, and in case of exposure to aerosols, of a filtering facepiece mask 2 or 3 (FFP2, FFP3) plus goggles or a face shield. On 3 March 2020 an outpatient SARS-CoV-2 RT-PCR-testing facility was opened at the Virchow campus. Designated testing sites for employees were established at all three campuses on 17 March 2020. Tests were analyzed in the routine laboratory work. Sampling for SARS-CoV-2 testing was done by a combined oro- and nasopharyngeal swab.

Continuous surveillance of SARS-CoV-2 was initiated in the beginning of March 2020. New cases of laboratory confirmed SARS-CoV-2 infections among patients and HCWs were evaluated by the IPC team. Contact persons, both patients and staff, with relevant exposure to infected individuals were identified. Contact tracing was conducted for a period of 48 h prior to the onset of symptoms, or 48 h before sample collection in case of asymptomatic infections. The definitions applied for contact tracing were oriented towards the SARS-CoV-2 contact categories by the German Robert Koch-Institute [16]. Contacts to SARS-CoV-2 positive persons were regarded as relevant for contact tracing if any of the following criteria applied:

- direct contact with potentially infectious body fluids (e.g. respiratory tract specimen) without proper PPE (patient to HCW contact),
- exposure to aerosols without proper PPE (patient to HCW contact),
- face-to-face contact under two meters without at least one person wearing a face mask (patient to patient, patient to HCW, HCW to patient and HCW to HCW contacts), or
- sharing the same patient room (patient to patient contact).

A healthcare-associated outbreak of SARS-CoV-2 infections was defined as two or more infections where an epidemiological link appeared likely, and where acquisition of the pathogen in the healthcare setting was assumed. Only laboratory-confirmed cases with a positive RT-PCR for SARS-CoV-2 were counted. We focused our analysis on the Virchow campus of the Charité-University Medicine hospital within a 2 month period (March and April 2020).

Hospitals in Germany are required by the German Protection against Infection Act to conduct continuous surveillance for healthcare-associated infections [17]. All data presented in this publication were collected in alignment with this regulation. Thus, ethical approval or informed consent were not necessary.

## Results

Between 1 March and 30 April 2020 we detected four healthcare-associated outbreaks of SARS-CoV-2 infections, comprising of 24 infected persons (23 HCWs and one patient). The outbreaks occurred in the departments of nephrology and dialysis (NEPH) (n = 9), anesthesiology (ANAE) (n = 8), surgical pediatrics (SPED) (n = 4), and neurology (NEUR) (n = 3).

Table 1 summarizes baseline characteristics and epidemiological key data of the NEPH outbreak. Case one was originally regarded as a single case that had returned from a vacation abroad on 15 March 2020, and that tested positive for SARS-CoV-2 2 days later. Case two was a relevant contact of case one and was tested positive for SARS-CoV-2 on 19 March 2020. As a result of the two positive tests, all relevant contacts and several other employees of the NEPH department underwent testing for SARS-CoV-2. Through this extensive testing, one patient and six other HCWs infected with SARS-CoV-2 were identified (cases 3–9 in Table 1). With the exception of case six, these all had relevant contact with either case one or two prior to the testing. None of the HCWs from the NEPH department had knowingly been involved in the treatment of COVID-19 patients before the outbreak. All patients with relevant contact to infected staff from the NEPH department were identified, isolated for 14 days after the last exposure and tested for SARS-CoV-2 if they were still hospitalized. Relevant contact patients already discharged, were immediately informed and instructed to isolate at home and contact health authorities if symptoms developed. No additional cases in the NEPH outbreak were noted after 23 March 2020.

**Table 1:** Baseline characteristics and epidemiological key data of the SARS-Coronavirus-2 (SARS-CoV-2) outbreak in the department of nephrology and dialysis

Case number	Function	Onset of symptoms	Date of sample collection for SARS-CoV-2 test	Relevant contact^a^ prior to onset of symptoms (or positive test if asymptomatic or unknown date of onset of symptoms)
1	Physician	15 March 2020	17 March 2020	None reported
2	Physician	18 March 2020	19 March 2020	Case 1
3	Physician	16 March 2020	20 March 2020	Cases 2, 9
4	Physician	21 March 2020	21 March 2020	Case 2
5	Patient	Unknown	23 March 2020	Case 2
6	Medical student	Unknown	23 March 2020	None reported
7	Medical student	Unknown	23 March 2020	Case 2
8	Nurse	Asymptomatic	23 March 2020	Cases 1, 4, 5
9	Physician	Asymptomatic	23 March 2020	Case 2

^a^Relevant contact was defined as direct contact with potentially infectious body fluids (e.g. respiratory tract specimen) without proper personal protective equipment (PPE) (patient to healthcare worker (HCW) contact), or exposure to aerosols without proper PPE (patient to HCW contact), or face-to-face contact under two meters without at least one person wearing a face mask (patient to patient, patient to HCW, HCW to patient and HCW to HCW contacts), or sharing the same patient room (patient to patient contact)

Table 2 illustrates key data of the ANAE outbreak. Cases one and two were initially considered isolated cases. Reportedly, no contact occurred between the two individuals. Neither of them had knowingly been involved in the treatment of COVID-19 patients prior to the positive tests. On 30 March 2020 case three was identified. Case three had been in direct contact (face-to-face contact without a face mask) with case number four of the NEPH outbreak. After identification of case three, all personnel that had been in contact with any of the first three cases of the ANAE outbreak, and several other employees of the ANAE department were tested for SARS-CoV-2. Through this process, five additional infections with SARS-CoV-2 were identified. Three of which (cases 4, 6, 7 in Table 2) reported having had relevant contact with other SARS-CoV-2 positive staff prior to testing. No nosocomial SARS-CoV-2 infections of patients occurred in the ANAE department, and no patients had been exposed to any of the infected HCWs without HCWs wearing medical masks. Cases 3, 4, 7, 8 worked in wards, in which COVID-19 patients or suspected cases were treated during the time of the outbreak. No contacts to COVID-19 patients without PPE were reported by any of the infected HCWs. The last case of the ANAE outbreak was recorded on 6 April 2020.

**Table 2:** Baseline characteristics and epidemiological key data of the SARS-Coronavirus-2 (SARS-CoV-2) outbreak in the department of anaesthesiology

Case number	Function	Onset of symptoms	Date of sample collection for SARS-CoV-2 test	Relevant contact^a^ prior to onset of symptoms (or positive test if asymptomatic or unknown date of onset of symptoms)
1	Nurse	Asymptomatic	20 March 2020	None reported
2	Physician	19 March 2020	23 March 2020	None reported
3	Physician	Unknown	30 March 2020	Case 4 of the nephrology outbreak
4	Physician	Unknown	3 April 2020	Case 3
5	Physician	31 March 2020	3 April 2020	None reported
6	Nurse	Asymptomatic	3 April 2020	Case 1
7	Nurse	Unknown	5 April 2020	Case 4
8	Nurse	Asymptomatic	6 April 2020	None reported

^a^Relevant contact was defined as direct contact with potentially infectious body fluids (e.g. respiratory tract specimen) without proper personal protective equipment (PPE) (patient to healthcare worker (HCW) contact), or exposure to aerosols without proper PPE (patient to HCW contact), or face-to-face contact under two meters without at least one person wearing a face mask (patient to patient, patient to HCW, HCW to patient and HCW to HCW contacts), or sharing the same patient room (patient to patient contact)

The SPED outbreak which consisted of four HCWs is displayed in Table 3. Case one was initially considered as an isolated case. After detection of case one, all relevant contact patients and employees were identified. Contact staff underwent testing for SARS-CoV-2. Contact patients still hospitalized were isolated for 14 days after the last exposure and tested for SARS-CoV-2. Patients already discharged, were informed and instructed to home isolate and contact health authorities if symptoms appeared. While none of the contact patients tested positive for SARS-CoV-2, three additional HCWs were identified by the extensive testing. Two of which (cases 2, 4 in Table 3) had been in relevant contact with case one prior to testing. None of the HCWs that tested positive for SARS-CoV-2 reported having been involved in the treatment of COVID-19 patients prior to testing. The last case of the SPED outbreak was detected on 3 April 2020.

**Table 3:** Baseline characteristics and epidemiological key data of the SARS-Coronavirus-2 (SARS-CoV-2) outbreak in the department of surgical paediatrics

Case number	Function	Onset of symptoms	Date of sample collection for SARS-CoV-2 test	Relevant contact^a^ prior to onset of symptoms (or positive test if asymptomatic or unknown date of onset of symptoms)
1	Physician	9 March 2020	23 March 2020	None reported
2	Physician	25 March 2020	25 March 2020	Case 1
3	Nurse	27 March 2020	29 March 2020	None reported
4	Nurse	31 March 2020	3 April 2020	Case 1

^a^Relevant contact was defined as direct contact with potentially infectious body fluids (e.g. respiratory tract specimen) without proper personal protective equipment (PPE) (patient to healthcare worker (HCW) contact), or exposure to aerosols without proper PPE (patient to HCW contact), or face-to-face contact under two meters without at least one person wearing a face mask (patient to patient, patient to HCW, HCW to patient and HCW to HCW contacts), or sharing the same patient room (patient to patient contact)

Details of the NEUR outbreak that consisted of three HCWs infected with SARS-CoV-2 are summarized in Table 4. Cases one and two tested positive for SARS-CoV-2 on consecutive days and had been in relevant contact in the days before. A third HCW with relevant contact to case one developed discreet symptoms of a respiratory tract infection and tested positive for SARS-CoV-2. Following the detection of the third case, staff with relevant contact to any of the cases underwent testing for SARS-CoV-2. Contact patients that were still hospitalized were isolated for 14 days after the last exposure and tested for SARS-CoV-2. Patients that were already discharged were instructed to isolate at home and contact health authorities in case symptoms developed. Testing of contact persons did not reveal any additional cases. The last case of the NEUR outbreak was detected on 30 March 2020.

**Table 4:** Baseline characteristics and epidemiological key data of the SARS-Coronavirus-2 (SARS-CoV-2) outbreak in the department of neurology

Case number	Function	Onset of symptoms	Date of sample collection for SARS-CoV-2 test	Relevant contact^a^ prior to onset of symptoms (or positive test if asymptomatic or unknown date of onset of symptoms)
1	Physician	13 March 2020	14 March 2020	None reported
2	Physician	14 March 2020	15 March 2020	Case 1
3	Physician	23 March 2020	30 March 2020	Case 1

^a^Relevant contact was defined as direct contact with potentially infectious body fluids (e.g. respiratory tract specimen) without proper personal protective equipment (PPE) (patient to healthcare worker (HCW) contact), or exposure to aerosols without proper PPE (patient to HCW contact), or face-to-face contact under two meters without at least one person wearing a face mask (patient to patient, patient to HCW, HCW to patient and HCW to HCW contacts), or sharing the same patient room (patient to patient contact)

In all four outbreaks, positive HCWs were exempt from work and sent into quarantine at home. Contact staff were allowed to continue working, but were instructed to closely monitor their health and continuously wear a face mask when in contact with patients or other personnel. In case of symptoms developing, contact HCWs were instructed to leave work immediately and undergo testing for SARS-CoV-2. The requirement to continuously wear a face mask was extended from contact staff to all HCWs in the hospital on 25 March 2020. All meetings had to be reduced to the minimum number of people necessary. Furthermore, HCWs were instructed to practice physical distancing during breaks and take meals separately. The public health authorities were notified of all four outbreaks. Outbreaks were considered contained if for a period of 28 days no new cases were noted. For all four outbreaks this was achieved.

## Discussion

Four outbreaks of SARS-CoV-2 infections in four different departments emerged at our campus within a short period of time. Two of the four outbreaks (NEPH and ANAE) were interlinked. With the exception of one patient in the NEPH outbreak, all infections occurred in HCWs. As the primary finding of our investigation we therefore conclude that HCW to HCW transmission can represent a critical factor in the spread of SARS-CoV-2 outbreaks in hospitals and may in some cases outweigh the risks posed by infected patients.

Interactions between HCWs in the hospital setting are frequent and an important aspect of a functioning team [18]. Physical distancing requires people to interact less or remotely and increase space between individuals [19]. The importance of physical distancing in the context of containment and mitigation of COVID-19 and other outbreaks has repeatedly been demonstrated [20, 21]. Despite heightened public awareness of the matter, our findings suggest that social interactions in our hospital initially continued without ensuring sufficient physical distancing. We consider the fact that the observed outbreaks almost exclusively consisted of HCWs and the multiple contacts between infected HCWs that were reported, a strong indicator that a majority of the observed SARS-CoV-2 infections were due to HCW to HCW transmission. This finding highlights the importance of incorporating a culture change towards strict physical distancing among colleagues, when not wearing a medical mask, into the practice of medicine during the time of the COVID-19 pandemic. Reports from other SARS-CoV-2 outbreaks in healthcare facilities that predominantly affected staff, reinforce this impression [15]. Molecular typing of the SARS-CoV-2 strains isolated from the infected individuals was not routinely performed at the time of the respective outbreaks and outbreak management was primarily focused on direct mitigation measures. As a result, meticulous reconstructions of chains of infections was not possible with the data available. Despite this limitation, we believe that the observed epidemiological constellation along with the decrease in cases after appropriate mitigation measures had been established, affirm our hypothesis of HCW to HCW transmission.

Steadily wearing a face mask can help reduce the spread of pathogens, including SARS-CoV-2 [22–24]. We believe that the most effective measure against the spread of SARS-CoV-2 at our campus was the decision to require all HCWs to continuously wear a medical face mask. This regulation did not only apply to direct patient care, but also to interactions with colleagues. It is remarkable that the large NEPH outbreak did not see any additional cases after the regulation to continuously wear face masks was put into practice on 25 March 2020. While cases occurred in the ANAE and SPED outbreaks after the regulation was established, daily observations indicated that incorporation of this new regulation into daily practice was not instantaneous, but took some time. Therefore, it is possible that some of the infections observed in the ANAE and SPED outbreaks that became apparent after 25 March, may have been acquired either before the date or during the “wash-in” period of universal face mask use. Another aspect that supports this interpretation is that no new outbreaks at out campus occurred during the observed period after the regulation to continuously wear face masks was put into effect.

Another observation was that unprotected contacts between HCWs often occurred in lunch and smoker breaks or in office situations, where masks were taken off and the physical distance between people was overestimated. The hospital IPC team frequently addressed this issue and emphasized the importance of continuous face mask wearing in office situations and recommended spending breaks and taking meals separately. Rising case numbers of SARS-CoV-2, both in the community and specifically among employees, might have also increased awareness on the matter and thereby facilitated adoption of mitigation measures by HCWs.

An aspect of similar importance for containing all four outbreaks was the identification and testing of personnel that had been exposed to SARS-CoV-2 carriers. Especially in the early stages of an outbreak it is crucial to quickly gain an overview about the number of cases. In this context, the long lag between the onset of symptoms and the testing of case one of the SPED cluster is noteworthy. Although speculative, it is conceivable that the other infections of the SPED cluster might have been avoided had case one been identified as SARS-CoV-2-positive sooner. The existence of a designated, readily accessible testing site for staff can facilitate rapid and systematic screening of HCWs. We believe that the employee testing site that commenced its operations on 17 March 2020 at our campus, was a key tool for successful outbreak management.

Various limitations have to be acknowledged when interpreting our findings. The COVID-19 pandemic is a dynamic situation with community-transmission occurring almost in all parts of the world. Therefore and due to the fact that molecular typing of the isolated SARS-CoV-2 strains was not performed, it is possible that some infections that were counted as part of the nosocomial clusters occurred outside the healthcare setting. Undetected SARS-CoV-2 infections among patients might have represented another source of infection. If such patients had been treated at our campus, it would have been possible that HCWs might have acquired the virus from patients rather than other HCWs. To the best of our knowledge, however, no such cases of undetected HCW exposure to COVID-19 patients occurred during the time of the reported outbreaks. Although the outbreak management was carried out in a sensitive and blame-free manner, some questions related to it may have been perceived as potentially compromising by some of the affected HCWs. Thus, not admitting to not using proper PPE or not acknowledging other unprotected contacts cannot be ruled out. Due to the possibility of false negative SARS-CoV-2 tests and the fact that not all HCWs in the entire hospital were tested, cases of SARS-CoV-2 infections in HCWs and potentially even outbreaks in other departments may have been missed. Compliance with measures, such as mandatory and universal wearing of face masks, might have varied between individuals. Therefore, the effect of such measures should be assessed with caution.

## Conclusions

PPE places a focus on protecting HCWs from infected patients. Undoubtedly, this represents a key aspect of reducing the spread of SARS-CoV-2 and maintaining HCWs’ health. The threat of infection from colleagues, however, might be underestimated by many HCWs. Increasing awareness of this potential source of infection, i.e. ensuring proper physical distancing measures and wearing of protective equipment when interacting with colleagues, must be a primary focus of infection prevention strategies in times of the COVID-19 pandemic. Since transmission of SARS-CoV-2 by asymptomatically infected individuals is possible, this aspect gains additional relevance.

## Data Availability

The datasets used and/or analyzed during the current study are available from the corresponding author upon reasonable request.

## Abbreviations

ANAE: Anesthesiology
COVID-19: Coronavirus disease 2019
HCW: Healthcare worker
IPC: Infection prevention and control
NEPH: Nephrology and dialysis
NEUR: Neurology
PPE: Personal protective equipment
RT-PCR: Reverse transcriptase polymerase chain reaction
SARS-CoV-2: Severe acute respiratory syndrome coronavirus 2
SPED: Surgical pediatrics
